# Kent and Kleemeier Award Lecture and Presentations

**DOI:** 10.1093/geroni/igaa057.3192

**Published:** 2020-12-16

**Authors:** Debra Dobbs

## Abstract

The Donald P. Kent Award lecture will feature an address by the 2019 Kent Award recipient, Terry Fulmer, PhD, of The John A. Hartford Foundation. The Kent Award is given annually to a member of The Gerontological Society of America who best exemplifies the highest standards of professional leadership in gerontology through teaching, service, and interpretation of gerontology to the larger society. The Robert W. Kleemeier Award lecture will feature an address by the 2019 Kleemeier Award recipient, Steven Zarit of Pennsylvania State University. The Kleemeier Award is given annually to a member of The Gerontological Society of America in recognition for outstanding research in the field of gerontology

